# Genetic Diversity of *Fusarium oxysporum* f. sp. *cubense*, the Fusarium Wilt Pathogen of Banana, in Ecuador

**DOI:** 10.3390/plants9091133

**Published:** 2020-09-01

**Authors:** Freddy Magdama, Lorena Monserrate-Maggi, Lizette Serrano, José García Onofre, María del Mar Jiménez-Gasco

**Affiliations:** 1 Facultad de Ciencias de la Vida, Escuela Superior Politécnica del Litoral, Campus Gustavo Galindo Km. 30.5 Vía Perimetral, Guayaquil 09015863, Ecuador; frearmag@espol.edu.ec; 2 Centro de Investigaciones Biotecnológicas del Ecuador, Escuela Superior Politécnica del Litoral, Campus Gustavo Galindo Km. 30.5 Vía Perimetral, Guayaquil E C090112, Ecuador; bmonserr@espol.edu.ec (L.M.-M.); lizveser@espol.edu.ec (L.S.); jangarci@espol.edu.ec (J.G.O.); 3 Department of Plant Pathology and Environmental Microbiology, The Pennsylvania State University, University Park, PA 16802, USA

**Keywords:** Fusarium wilt, banana, tropical race 4, clonal lineage, biosecurity, Ecuador

## Abstract

The continued dispersal of *Fusarium oxysporum* f. sp. *cubense* Tropical race 4 (*Foc*TR4), a quarantine soil-borne pathogen that kills banana, has placed this worldwide industry on alert and triggered enormous pressure on National Plant Protection (NPOs) agencies to limit new incursions. Accordingly, biosecurity plays an important role while long-term control strategies are developed. Aiming to strengthen the contingency response plan of Ecuador against *Foc*TR4, a population biology study—including phylogenetics, mating type, vegetative compatibility group (VCG), and pathogenicity testing—was performed on isolates affecting local bananas, presumably associated with race 1 of *F. oxysporum* f. sp. *cubense* (*Foc*). Our results revealed that *Foc* populations in Ecuador comprise a single clonal lineage, associated with VCG0120. The lack of diversity observed in *Foc* populations is consistent with a single introduction event from which secondary outbreaks originated. The predominance of VCG0120, together with previous reports of its presence in Latin America countries, suggests this group as the main cause of the devastating Fusarium wilt epidemics that occurred in the 1950s associated to the demise of ‘Gros Michel’ bananas in the region. The isolates sampled from Ecuador caused disease in cultivars that are susceptible to races 1 and 2 under greenhouse experiments, although Fusarium wilt symptoms in the field were only found in ‘Gros Michel’. Isolates belonging to the same VCG0120 have historically caused disease on Cavendish cultivars in the subtropics. Overall, this study shows how *Foc* can be easily dispersed to other areas if restriction of contaminated materials is not well enforced. We highlight the need of major efforts on awareness and monitoring campaigns to analyze suspected cases and to contain potential first introduction events of *Foc*TR4 in Ecuador.

## 1. Introduction

Bananas (*Musa* spp.), are widely cultivated in over 135 countries throughout tropical and subtropical regions and ranked as one of the most important crops in the world [1]. They represent an important source of calories with high nutritional value, contributing to an elevated intake of carbohydrates, fiber, vitamins, and minerals [2] in the diet of over 500 million people [3]. In some countries in East Africa, including Uganda, Rwanda, and Cameroon, the annual consumption of bananas may exceed 200 kg per capita [4]. Banana plants are also used as raw materials in a wide range of domestic and regional products [3] and serve as cash crop for smallholder farmers.

Among the total annual world production of bananas, from the 153 million tons reached in 2017 [5], only 15% accounts for international trade, whereas the remaining product is destined for local consumption [6]. Notably, the export trade of bananas makes a large contribution to national economies of main exporting countries, such as Ecuador, where bananas and their derived products represent the second largest export commodity after petroleum [4]. It should be noted only 3 of the 50 recognized subgroups of banana account for 75% of the current global production [7]. This includes the AAA Cavendish cultivars, the AAB plantain subgroup, and the ABB East African Highland bananas [8].

Diseases are serious constraints to banana production, both for export and domestic markets [6]. Among them, Fusarium wilt of banana (FWB) is currently the most severe threat for the banana industry worldwide. This disease is caused by the soil-borne fungus *Fusarium oxysporum* f. sp. *cubense* (*Foc*), the pathogen that caused the demise of ‘Gros Michel’, a previous cultivar used in the export industry in Central and South America. There is no chemical control for FWB and there are limited other options for managing this disease. This global risk is exacerbated by many factors, including the monoculture of bananas and its genetic uniformity that creates suitable scenarios for the development of epidemics. In addition, socio-economic factors, including weak national phytosanitary agencies and regulations, poor surveillance systems, lack of awareness and allocation of funding for national research programs, non-articulated contingency plans, restrictive laws for the adoption of new breeding technologies, etc., are additional hurdles that impede progress. Currently there are 24 Vegetative Compatibility Groups (VCGs) of *Foc* distributed in clonal lineages [9], and three well-differentiated pathogenic races [10] found worldwide. VCGs serve as a natural means to subdivide a fungal species based on the premise that isolates in the same VCG can anastomose to form a stable heterokaryon and are presumed to be identical at all of the *vic* loci [11]. Among all VCGs, VCG-01213/16 comprises the group of strains referred to as Tropical race 4 (TR4). This race is jeopardizing the entire export industry based on cultivars of the Cavendish subgroup, as well as, and of equal relevance, the livelihood of millions relying on a wide range of other susceptible varieties in Oceania, Asia, India, Africa, and Latin America [12]. TR4 differs from races 1, 2, and subtropical race 4 (STR4), because it can affect a wider range of banana cultivars and cause disease to Cavendish in tropical environments, in the absence of predisposing conditions [10]. Since its emergence in 1990 in Southeast Asia, TR4 has spread to Western Asia, Africa, and most recently, South America. As of now, TR4 is reported in all continents, and specifically in the following countries, Taiwan, Indonesia, Malaysia, China, Australia, Philippines, Oman, Jordan, Mozambique, India, Pakistan, Lebanon, Israel, Vietnam, Myanmar, Laos, England, Colombia, Turkey, and the island of Mayotte (France) [9,13,14,15,16,17,18,19,20].

In the 1950s, *Foc* destroyed nearly 100,000 hectares of ‘Gros Michel’ banana plantations in Central and South America, with an estimated loss of over USD $2 billion [13]. Current losses by TR4 in Indonesia, Taiwan, and Malaysia amount to USD $388 million [21], although greater losses are projected in other significant Cavendish-producing countries, such as China and the Philippines [13]. Recent research predicts that 13.1–17.7% of the world area dedicated to banana production will be lost over the next 25 years, depending on the spread rate of TR4 [22]. In Australia, industry losses are predicted to exceed USD $138 million per year considering a slow rate of spread of the pathogen, [23].

Ecuador is the world’s largest exporter of Cavendish banana. In 2018, 6.4 M tons of bananas were exported, with an estimated FOB value of USD $2999 million [24]. FWB was first observed in Ecuador in 1936 in the Guayas province [25], although national records suggest it could have occurred as early as 1929. The replacement of the susceptible ‘Gros Michel’ by Cavendish cultivars, which took place as a large-scale management strategy in intensively grown banana plantations, eliminated the awareness of Fusarium wilt threats. However, the disease continues to affect small-holder farmers that still cultivate ‘Gros Michel’ and other bananas, such as ‘Manzano’, grown mainly for family subsistence and local markets. To date, no information exists on the distribution, population structure, race identity, or genetic diversity of *Foc* populations in Ecuador.

Various VCGs, including 0120/15, 0121, 0124, 0125, 0126, 0128, 01210, and 01214, have been reported in several countries in the Americas and Caribbean [26,27,28,29,30,31,32,33,34,35]. We hypothesize that similar genotypes to those mentioned above will be found in Ecuador, reflecting the movement of planting material and trade among Central and South American countries. Although information regarding *Foc* populations from countries neighboring Ecuador is incomplete, a recent assessment in Peru about the incidence of FWB in different production systems found eight different banana and plantain varieties affected by FWB [36], suggesting the presence of more than one *Foc* VCG. Also, besides the recent incursion of TR4 in Colombia, not much is known about the pathogen in that country [18].

The study of pathogen populations is an essential step for the deployment of suitable management strategies, including selection of resistant cultivars, development of diagnostic tools, and implementation of quarantine regulations across borders [37]. Studying the current populations of *Foc* can also shed light on how these strains can serve as reservoirs for virulence genes that can lead to emergence of new pathogenic variants, either towards other banana cultivars, or other crops [38,39]. The main aim of this research was to evaluate the genetic diversity of *Foc* from the main banana-producing provinces of Ecuador by means of pathogenicity testing, VCG testing, and genetic characterization.

## 2. Results

### 2.1. Distribution of Fusarium Wilt in Ecuador and Fungal Sampling

**Figure 1:**
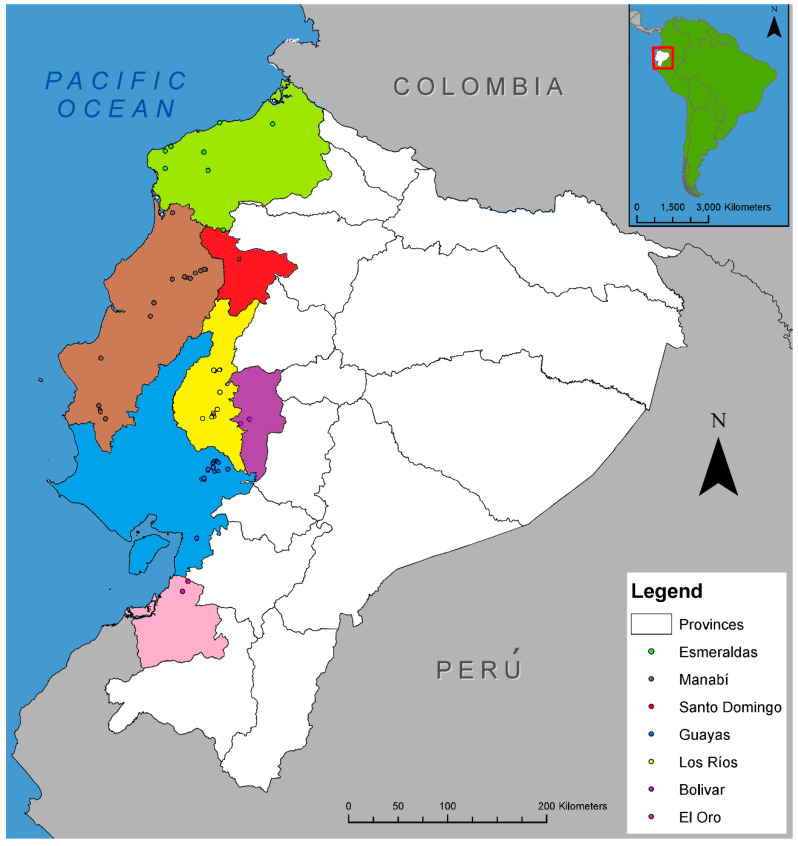
Distribution map showing sampling sites of Fusarium wilt of banana observed across the coastal area of Ecuador. Colored dots represent locations visited in seven provinces of Ecuador (Esmeraldas, Manabí, Guayas, Los Ríos, El Oro, Bolívar, and Santo Domingo).

**Figure 2:**
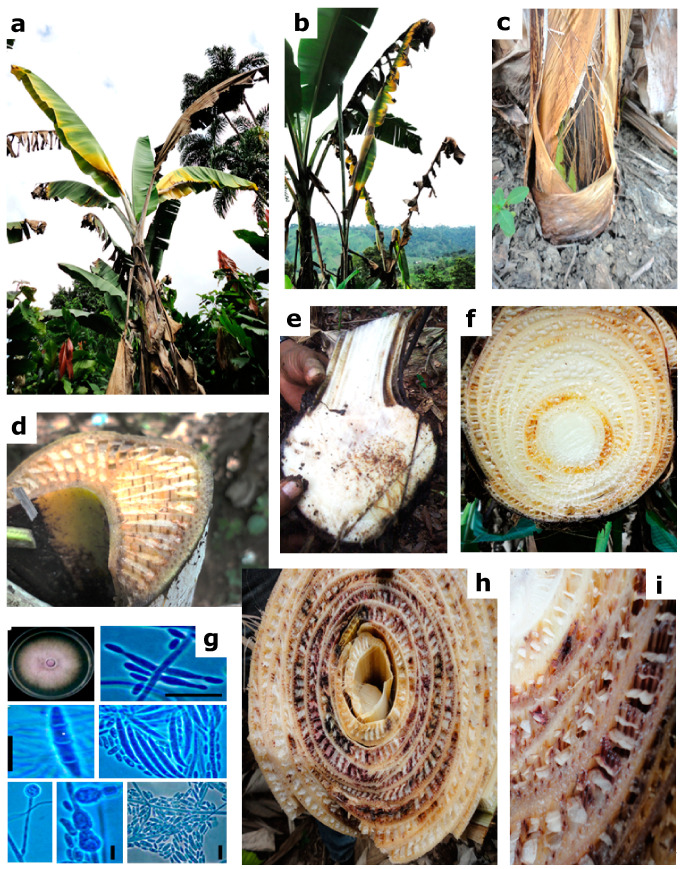
Symptoms and signs associated with Fusarium wilt of banana: (**a**) Yellowing and wilting of leaves. (**b**) Rolling of young leaves. (**c**) Cracking of pseudo-stem. (**d**) Fungal invasion of veins. (**e**) Infected rhizome. (**f**) Early stage of vascular infection. (**g**) Morphological features and types of spores of *Fusarium oxysporum* f. sp. *cubense*. (**h**,**i**) Advanced stages of vascular infection with typical reddish-brown discoloration.

FWB was present in all provinces from the coastal area of Ecuador surveyed, including Esmeraldas, Manabí, Guayas, Los Ríos, El Oro, Bolivar, and Santo Domingo (Figure 1). A total of 291 isolates of *F. oxysporum,* initially identified based on morphological characters, were obtained from infected tissue of ‘Gros Michel’ banana plants showing typical symptoms of FWB; that is, wilting, yellowing of leaves, splitting of the pseudostem, and vascular necrosis (Figure 2). Banana plants from other cultivars, such us ‘Manzano’ (a.k.a. ‘Apple’), ‘4 Filos’ (a.k.a. ‘Bluggoe’), ‘Maqueño’, ‘Orito’ (a.k.a. ‘Lady Finger’), ‘Morado’ (a.k.a. ‘Red’), Cavendish cultivars, and plantain ‘Barraganete’, were not found showing symptoms of FWB during our survey. However, seven putative nonpathogenic (endophytic) isolates of *F. oxysporum* obtained from the roots of asymptomatic banana plants were incorporated into the analyses. Those isolates were EC1-LR-CV1 and EC4-LR-CV1 (from Cavendish), EC5-LR-GM1 and EC10-B-GM3 (from ‘Gros Michel’), EC7-LR-PL1 and EC8-LR-PL2 (from ‘Barragete’), and EC11-B-OR2 (from ‘Orito’). In total, 298 isolates were used in this study.

### 2.2. Identification of Fusarium oxysporum and Phylogenetic Analyses

**Figure 3:**
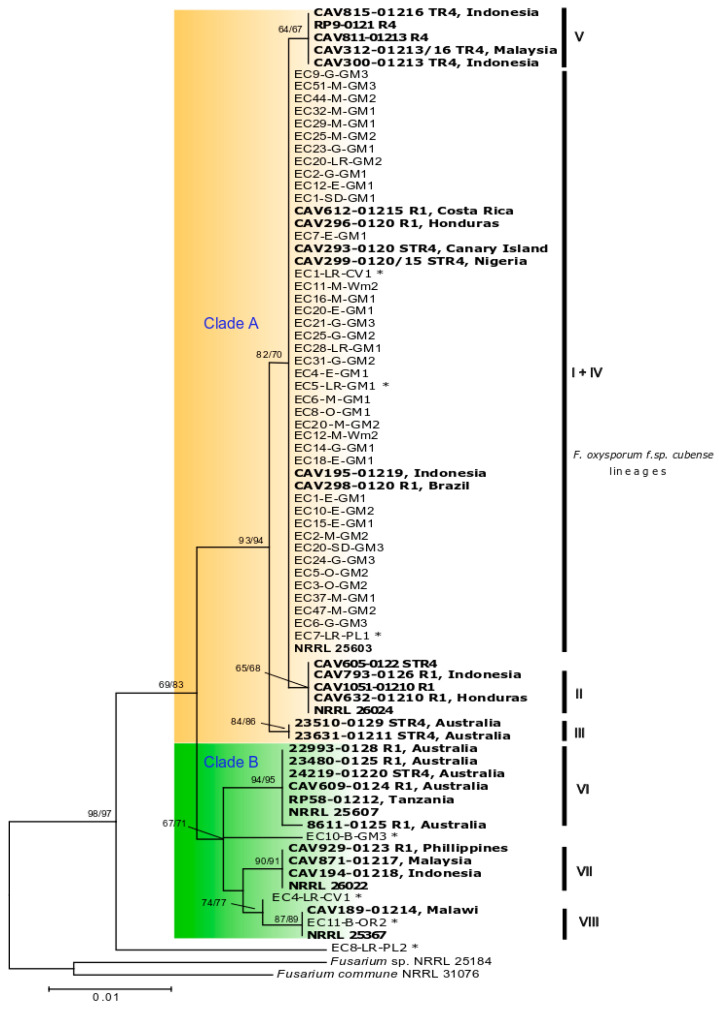
Maximum likelihood phylogenetic tree containing 44 selected *F. oxysporum* isolates associated to banana from Ecuador inferred from the TEF region sequence data. Clades are color-differentiated and included the various *F. oxysporum* f. sp. *cubense* strains (marked in bold) described by Fourie et al. [10] and indicated to the right side of the tree as lineages (I to VIII). Numbers above internodes represent the ML and MP bootstrap support. *F. oxysporum* from asymptomatic banana are marked with an *. The tree is rooted with *Fusarium* sp. NRRL 25184 and *Fusarium* commune NRRL 31076.

Following morphological and microscopic analyses, sequencing results from the TEF region (625 bp) confirmed all 298 isolates as *F. oxysporum*. The first phylogenetic analysis, which included sequences for 233 isolates, showed that most isolates shared identical TEF sequences (Supplementary Materials, Figure S1). Based on this result we selected 44 representative isolates for further analysis based on the IGS region (1332 bp). Phylogenetic analysis of TEF sequences placed most isolates from Ecuador in a single group within clade ‘A’ that contained lineages I and IV described by Fourie et al. [10] and VCGs 0120, 01215 (lineage IV), and 01219 (lineage I) (Figure 3). Also, isolates obtained from the roots and rhizomes of asymptomatic Cavendish, ‘Gros Michel’, and ‘Barraganete’ banana plants, namely EC1-LR-CV1, EC5-LR-GM1, EC7-LR-PL1, were also placed within the I+IV lineage. The only isolates placed in clade ‘B’ *sensu* Fourie et al. [10], were EC10-B-GM3, EC4-LR-CV1, and EC11-B-OR2, obtained from asymptomatic ‘Gros Michel’, Cavendish ‘Williams’, and ‘Orito’ banana plants, respectively. Among them, only the isolate EC11-B-OR2 presented a TEF sequence similar to the strains CAV189 and NRRL25367, associated with VCG01214 and described as lineage VIII of *Foc*. In addition, the isolate EC8-LR-PL2, obtained from the roots of an asymptomatic ‘Barraganete’ banana was the only one not included either in clade A or B, where all other lineages of *Foc* were found.

**Figure 4:**
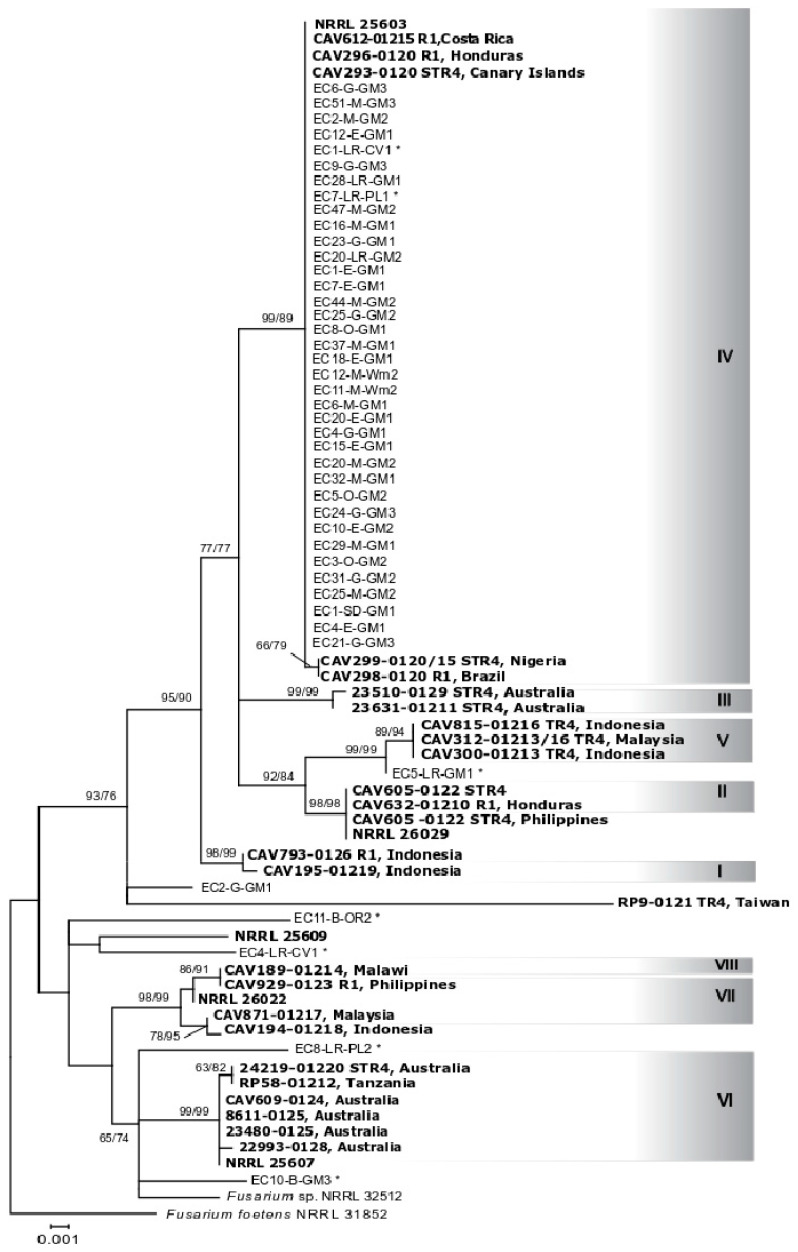
Maximum likelihood phylogenetic tree containing 44 selected *F. oxysporum* isolates associated to banana from Ecuador inferred from intergenic spacer region (IGS) sequence data. Clonal lineages previously identified in translation elongation factor 1 alpha (TEF)-maximum likelihood (ML) tree are shadowed and reference strains marked in bold. *F. oxysporum* isolates from asymptomatic banana are marked with an *. Numbers above internodes represent the ML and maximum parsimony (MP) bootstrap support. The tree is rooted with *Fusarium* sp. NRRL 32512 and *Fusarium foetens* NRRL 31852.

Phylogenetic analysis of the IGS region from the same 44 selected isolates from Ecuador generated a tree with a similar topology (Figure 4). Almost all isolates that were placed in a single TEF clade, but two, grouped also in a single lineage (IV), genetically similar to reference strains CAV612, CAV296, CAV293, CAV298, and CAV299, from Costa Rica, Honduras, Spain, Brazil, and Nigeria, belonging to VCGs 0120 and 01215. The isolates EC5-LR-GM1 (obtained from an asymptomatic plant) and EC2-G-GM1 were the only exceptions not included in this lineage. Furthermore, isolate EC5-LR-GM1 was closely included in a subgroup of isolates reported as TR4, belonging to VCG01213 and 01216. The IGS region provided further resolution to differentiate the isolates from Ecuador from VCG01219, represented in this analysis with the strain CAV195, which was part of the lineage I + IV of *Foc* based on the TEF phylogeny. As per the endophytic isolates included, their phylogenetic placement was similar to that defined by TEF.

### 2.3. Identification of Mating Types and VCG Determination

Amplification of genomic DNA with MAT1-2 specific primers (FF1 and Gfmat2c) generated a 700 bp single band for 294 (98.7%) of the 298 isolates tested (Supplementary Materials, Table S1; Figure S2), including isolates EC1-LR-CV1 obtained from asymptomatic Cavendish ‘Williams’ banana plant, and EC11-B-OR1 and EC11-B-OR2 from ‘Orito’ banana. PCR reactions using genomic DNA from the same isolates as a template with specific primers for MAT1-1 (Falpha 1 and Falpha 2) failed to produce the expected 370-bp amplicon. Isolates EC4-LR-CV1, EC5-LR-GM1, EC7-LR-PL1, and EC8-LR-PL2 were the only ones that generated an amplicon for MAT1-1.

**Figure 5:**
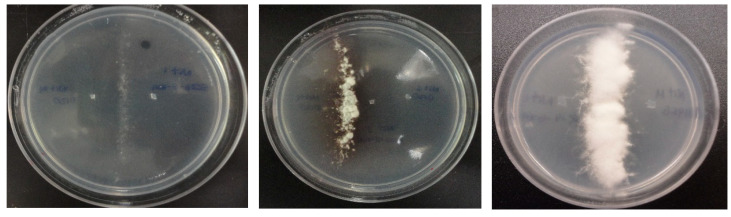
Categorical illustration of the different heterokaryon formation during compatibility tests using *Nit*-mutants: Weak compatibility (**left figure**), moderate compatibility (**central figure**), strong compatibility (**right figure**).

*Nit* mutants were successfully generated from 23 isolates and then paired with the three tester strains from the VCGs used (VCG0120, VCG0123, and VCG0123) (Table 1). *Nit-1* and *Nit-3* mutants of 18 isolates from Ecuador generated stable heterokaryons when paired with *Nit-M* mutants of VCG0120, however not all of them generated a strong heterokaryon, and sometimes the complementation was moderate or weak (Figure 5). Five isolates failed to form a heterokaryon. On the other hand, no compatibility was observed when *Nit1*-or *Nit-3* mutants of the tested isolates were paired with *nit-M* mutants of VCGs 0123 and 01213 tester strains.

### 2.4. Pathogenicity Test and Race Determination

**Figure 6:**
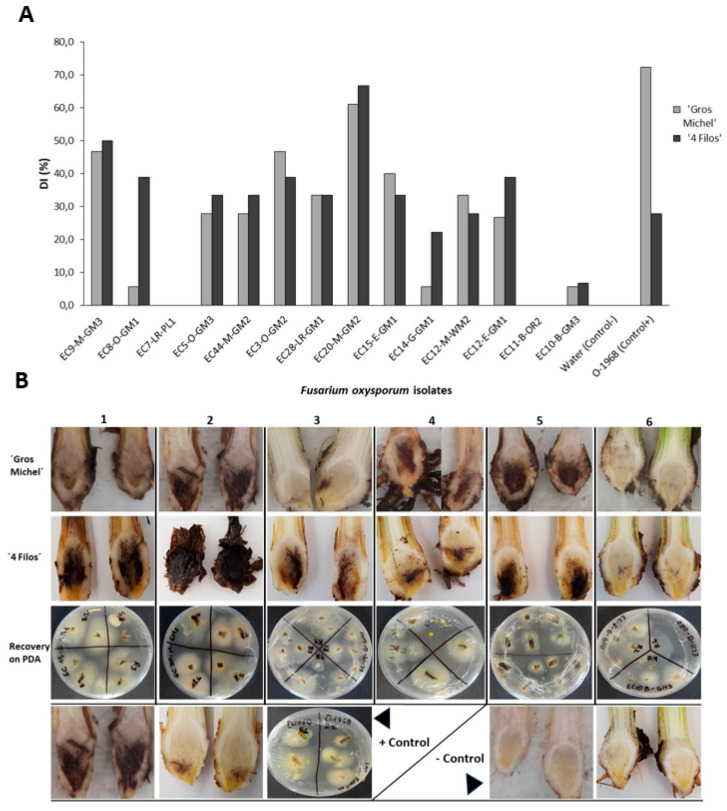
Differential response of ‘Gros Michel’ and ‘4 Filos’ banana plants 45 days post-inoculation with *Fusarium oxysporum* isolates from Ecuador using corn-meal sand inoculation. (**A**) Disease index (DI) according to McKinney infection index [40]. (**B**) Internal symptoms of Fusarium wilt of banana (FWB) caused by six isolates representative of each province sampled inoculated on two banana cultivars: 1 = EC15-E-GM1, 2 = EC20-M-GM2, 3 = EC14-G-GM1, 4 = EC28-LR-GM1, 5 = EC3-O-GM2, 6 = EC10-B-GM3 and, recovering of *F. oxysporum* from rhizome-affected tissues.

Based on an internal symptom severity rating scale, most isolates were pathogenic on either race 1- or race 2-susceptible cultivars. Among the 14 isolates inoculated on ‘Gros Michel’and ‘4 Filos’ banana plants, 12 caused typical symptoms of Fusarium wilt. Severity values for internal symptoms were isolate-dependent, presenting DI values ranging from 5.6 to 61.1% for ‘Gros Michel’ and from 6.7 to 66.7 for ‘4 Filos’ (Figure 6A). Among all, isolates EC9-M-GM3, EC3-O-GM2, and EC20-M-GM2 presented higher disease index values, whereas isolates EC10-B-GM3 and EC14-G-GM1 were the ones with the lowest. Conversely, no isolate of *F. oxysporum,* out of the 45 inoculated on Cavendish ‘Williams’ banana induced symptoms of wilting or rhizome necrosis even 45 days post inoculation. We included three of the isolates sampled from asymptomatic plants, EC7-LR-PL1, EC10-B-GM3, and EC11-B-OR2. Isolates EC7-LR-PL1 and EC11-B-OR2 were the only ones that did not cause disease on any cultivar evaluated (Figure 6B). No symptoms were observed in non-inoculated banana plants, and *F. oxysporum* colonies were recovered from randomly sampled symptomatic banana-rhizomes at the end of the experiment. Reference strain O-1968 (VCG 0123) used as control presented higher PDI values on ‘Gros Michel’ than on ‘4 Filos’.

## 3. Discussion

Bananas feed more people per unit area of production than any other staple crop. Cultivation of banana worldwide relies on a few cultivars. Most of the banana produced for export in Central and South America depends on the extensive and intensive use of cultivars in the Cavendish group (such as ‘Williams’, ‘Gran Naine’, etc.), which creates an ideal scenario for pathogens and pest to affect banana production severely affecting entire fields. This is the case for *Foc* TR4 a pathogen that emerged in South East Asia and currently poses the biggest threat to all banana-producing countries, either for local consumption or export, as it kills a great diversity of banana cultivars and contaminates soil for decades [41].

We searched for FWB in seven provinces across the coastal area of Ecuador where most banana production takes place, and found the disease occurring exclusively in ‘Gros Michel’. Contrary to our initial hypothesis, our results indicate that *Foc* populations in Ecuador comprise a single clonal lineage. Its wide distribution across the major banana-producing provinces in the coastal area of the country may have occurred by the movement of infected material, such as suckers or soil from contaminated farms to other pathogen-free areas. In this study, we conducted phylogenetic analyses based on TEF and IGS regions, loci that have been frequently used for inferring phylogenetics and identification of *F. oxysporum,* and for the study of pathogenic and nonpathogenic populations [42,43,44,45,46]. We compared isolates sampled in Ecuador with those representing a worldwide collection of *Foc* previously studied [10]. All isolates sampled from symptomatic bananas shared identical TEF and IGS sequences (Figure 3 and Figure 4, Supplementary Figure S1). The TEF phylogeny placed isolates with reference strains of VCGs 0120, 01215 (lineage IV), and 01219 (lineage I) [10]. The IGS region further differentiated isolates from Ecuador from the reference strain CAV195-VCG01219 (lineage I), previously grouped with VCGs 0120 and 01215 (lineage IV) in the TEF analysis. Although lack of congruency between TEF and IGS has been well documented in prior studies [10,44], the analyses of both regions suggested that isolates from Ecuador share a single common ancestor. The presence of only one mating type (MAT1-2 idiomorph) in all *F. oxysporum* isolates obtained from symptomatic banana (N = 291) is consistent with the presence of a single clonal lineage. In line with previous reports, none of the isolates analyzed presented both idiomorphs [47].

Only one VCG (0120) was found in samples tested from Ecuador (Table 1), supporting the idea of a single clonal lineage. Cross compatibility between VCGs 0120 and 01215 has been documented, so further tests including VCG01215 should be conducted [10]. Pathogenicity tests using Cavendish ‘Williams’, ‘Gros Michel’, and ‘4 Filos’ showed that most isolates tested caused symptoms on race 1- and race 2-susceptible cultivars under greenhouse conditions, however only a race 1 phenotype was observed in the field as no cultivar other than ‘Gros Michel’ presented Fusarium wilt symptoms. This was constant in all provinces sampled, although average weather (precipitation and temperature) deviated from the normal records during the month prior to sampling (April, 2013), according to the National Institute of Meteorology and Hydrology (INAMHI). Increased precipitation values were recorded in Esmeraldas (11%), Manabí (119%), Santo Domingo and Los Ríos (16%), whereas decreased values were recorded in Guayas (−37%) and El Oro (−82%). Similarly, higher temperature was recorded in Esmeraldas (22.6–31.6 °C), Santo Domingo (20.4–30.5 °C), Los Ríos (21.9–33.8 °C), and El Oro (21.6–34.4 °C), while lower temperatures occurred in Manabí (18.3–34.2 °C) and Guayas (21.8–33.5 °C). We acknowledge that race structure in *Foc* is confusing and often inaccurate in delineating strains [48]. Our results are in accord with previous reports as VCG120 has not been described affecting ‘Bluggoe’ (‘4 Filos’) bananas in the field, and is more commonly found in symptomatic ‘Gros Michel’ (AAA) and other cultivars like ‘Silk’ (AAB), ‘Pome’ (AAB), ‘Pisang Awak’ (ABB), and ‘Maqueño’ [30]. The susceptibility shown by ‘4 Filos’ to VCG0120 in the pathogenicity experiment might be associated to the age of plantlets used, temperature, or inoculum load [10]. The consumption of ‘4 Filos’ banana in Ecuador is limited, grown in small plots in few rural areas. Our results indicate the need for field testing when searching for resistance against FWB in local varieties or breeding programs. Interestingly, VCG0120 has also been associated with the subtropical race 4, causing disease on Cavendish cultivars under stress conditions in the subtropics [13,49]. It is not clear whether the different phenotypes observed with isolates of this VCG are somewhat associated to certain genetic variants or truly dependent on environmental factors. If the last notion is true, it may explain the occurrence of Fusarium wilt symptoms on Cavendish cultivars in the state of Santa Catalina (Brazil) where temperatures in the southern area of the state are lower, mainly in the winter (average minimum temperature of 14.2 °C, compared to 17.0 °C in the north). [33]. Overall, this study has shown that the isolates causing FWB in Ecuador analyzed in this study comprise a single clonal lineage. Results of this study also discarded the presence of *Foc*TR4 in Ecuador as none of the isolates from symptomatic bananas evaluated were phylogenetically similar to VCG01213 or were vegetatively compatible with the TR4 tester strain O-2052 used in this study.

We also studied *F. oxysporum* endophytic isolates sampled from asymptomatic banana plants. The TEF phylogeny placed isolates EC1-LR-CV1, EC5-LR-GM1, EC7-LR-PL1, and EC11-B-OR2 in clades that included strains pathogenic to banana (Figure 3). Isolates EC1-LR-CV1 and EC7-LR-PL1 shared identical TEF and IGS sequences with isolates pathogenic to banana, but EC7-LR-PL1 did not cause any disease when inoculated on Cavendish ‘Williams’, ‘Gros Michel’, and ‘4 Filos’, and the pathogenicity of EC1-LR-GM1 was not evaluated. Isolate EC11-B-OR2 was placed with CAV189 (VCG01214) by TEF, although unresolved by IGS, and did not cause any disease in pathogenicity tests. EC5-LR-GM1 shared identical TEF sequences with pathogenic isolates from Ecuador in lineage I + IV; but it is closely related to TR4 based on IGS sequences. Previous research showed that EC5-LR-GM1 is not pathogenic to Cavendish ‘Gran Naine’ and ‘Gros Michel’, but along with other endophytes, it shares genomic regions similar to those found in TR4 strains that have been exploited for PCR diagnostics leading to false positives [50]. Another interesting isolate was EC10-B-GM3, sampled from asymptomatic ‘Gros Michel’, with unresolved phylogenetic placement. This isolate caused a certain level of rhizome necrosis, although of lower symptom severity compared to the disease caused by isolates in VCG0120. This necrosis could result from the high inoculum levels used in pathogenicity testing but raises the possibility of isolates colonizing plants in the field asymptomatically but possess pathogenic potential. Several studies showed that within the *F. oxysporum* species complex, greater diversity is found in endophytes and soil-derived isolates than in their pathogenic counterparts [51,52]. Thus, it is not uncommon to find nonpathogenic isolates associated with pathogenic forms. In fact, Fourie et al. [10] reported that isolates associated with VCG01214 were more closely related to nonpathogenic isolates and other *formae speciales* of *F. oxysporum* than to other lineages of *Foc*. It is not known whether the endophytes found in this study were introduced in conjunction with pathogen populations when bananas were introduced into the country or are part of the native population.

The 1950s decimation of ‘Gros Michel’ in Central and South America has been assumed to have been caused by individuals in a single VCG [53]. Early strains from the Americas (Costa Rica, Honduras) have been placed in VCG0120, the same VCG that contains current isolates from Ecuador. To date, we know this VCG is present in Costa Rica, Honduras, Brazil [26], Peru [54], and Ecuador (this study). In Venezuela, the clonal lineage VCG01215 was reported in the state of Trujillo [26]. We speculate the populations of *Foc* currently found in Ecuador are remnants of the main group of *Foc* lineages that caused the 1950s epidemics. Sampling was done on backyards, home-gardens, roadsides, abandoned farms, cacao plots, and small banana farms, from cultivars that are mainly used for local consumption (which were the only ones displaying symptoms), so it is not unlikely that agricultural trade has not been so intense in this context, especially regarding international trade. No disease was found in large Cavendish intensive plantations grown for export. We can conclude that one clonal lineage is found in Ecuador and it seems to be widely established in the country. That same lineage (VCG0120) contains strains sampled in the 1950s in other South and Central American countries, and it is presumed to be the main lineage that resulted in the destruction of ‘Gros Michel’. Whether current *Foc* populations in Ecuador are the result of one introduction event, or multiple introductions of the same clonal lineage from neighboring countries is hard to test and would require more powerful molecular makers. However, given the lack of lineage diversity of *Foc* found in Ecuador compared to the high lineage diversity of *Foc* found worldwide, including other American countries, we can speculate that movement of the pathogen into Ecuador has been limited. It is not clear how *Foc* was introduced into Ecuador, but there are two potential scenarios. The first possibility is that *Foc* was introduced before ‘Gros Michel’ and Cavendish were adopted. Historical records indicate that bananas were already present in several countries of Central and South America, including Ecuador, before the arrival of the Spaniards [55]. Langdon [56] suggested that bananas were a major food crop in both Polynesia and tropical South America for over 2000 years. In a second scenario, bananas were first introduced into the New World in 1516 by Father de Berlanga [57], and it is plausible, that *Foc* was introduced after that, along the trade routes of ‘Gros Michel’ from Martinique to Jamaica in 1835, and from this point further dispersed by Dutch, British, French, and German traders [57,58]. In Ecuador, the banana trade did not begin until 1910 [55]; however, Colombia made its first import of ‘Gros Michel’ banana plants from Jamaica in 1892 [58]. In 1934, the United Fruit Company (now Chiquita), fleeing banana diseases and labor arrest in Central America, made its way to South America passing from Colombia to finally settle in Ecuador [59]. Whether *Foc* was introduced into Ecuador directly from Jamaica or other Central America countries like Costa Rica or Panama, or from a neighbor country, still remains unknown.

The baseline knowledge generated in this study can enlighten the deployment of better management strategies considering the current dependence on a single dominant group of bananas, the Cavendish. Although market-desirable, it represents a perfect formula for pathogens and pests to affect banana production. Major emphasis should be given to surveillance and the strict control on the movement of plant material in order to avoid the introduction, or in the worst-case scenario, the dispersal of new pathogens, such as TR4. For Ecuador, TR4 could become a real management challenge considering the size of its banana industry, which comprises approximately 173,706 ha of Cavendish cultivars, 125,268 ha of plantain, and 3742 ha of sucrier banana ‘Orito’, according to official data from 2018 [60]. Concerns have been raised since the incursion of the pathogen into the neighboring country of Colombia, the first report of TR4 in South America [18]. In June 2019, two banana farms in the department of Guajira, northeast of Colombia, reported symptoms of Fusarium wilt [61]. So far, nearly 175 ha of banana farms have been eradicated and are under surveillance by the Colombian Agricultural Institute (ICA), the country’s federal agency tasked with overseeing agricultural health [61]. For this reason, exclusion measures have gained significant importance in Ecuador to minimize the risk of introduction, including the accomplishment of a contingency plan manual, harmonizing diagnostic protocols among laboratories, building up a legal framework, and increasing awareness trough educational campaigns supported by the National Plant Protection Organization (AGROCALIDAD) and the private sector. However, efforts to develop resistant bananas for TR4 and the implementation of biosecurity measures from country borders to farm gates continue to be cumbersome.

## 4. Materials and Methods

### 4.1. Sampling and Fungal Isolation

*Fusarium oxysporum* isolates were obtained from banana plants displaying symptoms of Fusarium wilt from several provinces in the coastal area of Ecuador, including Esmeraldas, Manabí, Guayas, Los Rios, El Oro, Santo Domingo, and Bolivar (Figure 1). The search for symptomatic plants followed the main routes connecting the provinces mentioned above. Sampling sites included backyards, home-gardens, roadsides, abandoned farms, cacao plots, and small banana farms.

Samples of necrotic tissue showing a reddish-brown discoloration were collected from the pseudostem of plants. Samples were processed under aseptic conditions, surface-disinfested with 70% ethanol for 30 s and 5% sodium hypochlorite for one minute, dried in sterile filter paper after rinsed with autoclaved water, placed in plates containing Nash *Fusarium* semi-selective medium [62], and incubated at 28 °C for five days. Growing colonies were transferred to Potato Dextrose Agar (PDA) plates containing 120 mg/mL of streptomycin. Single-spore colonies were obtained for each isolate, and sub-cultured for short-term use and long-term preservation. For long-term preservation, five plugs of media containing mycelia, per isolate, were transferred into 1 mL crio-vials containing a 1:1 (vol/vol) solution made of half-strength Potato Dextrose Broth (PDB) and 75% glycerol, and kept at −80 °C. The entire collection was deposited in the Culture Collection of Microorganisms (CCM-CIBE) of the Biotechnological Research Center of Ecuador, and a copy at the Pennsylvania State University. Isolates were initially characterized as *Fusarium* spp. based on the colony morphology and the different types of conidia (including micro and macroconidia) observed under the microscope.

### 4.2. DNA Extraction

Isolates of *Fusarium* spp. were grown in PDB for 5–7 days at 28 °C. Mycelium was harvested and dried using sterilized filter paper, followed by DNA extraction using a protocol derived from Cenis [63], and commercial kits (Qiagen, Valencia, CA, USA) according to the manufacturer’s instructions. DNA concentration and quality were estimated using a Nanodrop spectrophotometer-2000 (Thermo Fisher Scientific, Wilmington, DE, USA) followed by gel electrophoresis.

### 4.3. PCR Amplification, Sequencing, and Phylogenetic Analyses

Two regions were used for phylogenetic analyses, a partial region of the translation elongation factor 1 alpha (TEF) gene and the intergenic spacer region (IGS) of the ribosomal DNA. The TEF region was amplified using the primers ef1 (5’-ATGGGTAAGGARGACAAGAC-3’) and ef2 (5’-GGARGTACCAGTSATCATGTT-3’) [43], and the IGS region using the primers CNL12 (5’-CTGAACGCCTCTAAGTCAG-3’) and CNS1 (5’-GAGACAAGCATATGACTACTG-3’) according to Apple and Gordon [64]. PCR reactions were carried out in 25 μL using Choice Taq Master Mix (Denville Scientific Inc., Holliston, MA, USA), 0.5 μM of each primer, 10–20 ng of DNA and sterile deionized water. The cycling conditions for amplification of the TEF region were as follows: Initial denaturation at 94 °C for 2 min, followed by 35 cycles of denaturation at 94 °C for 45 s, annealing at 60 °C for 45 s, elongation at 72 °C for 1 min 30 s, and a final extension at 72 °C for 5 min. The cycling conditions for amplification of the IGS region were as follows: Initial denaturation at 94 °C for 5 min, followed by 30 cycles of denaturation at 94 °C for 1 min, annealing at 54 °C for 1 min, elongation at 72 °C for 1 min, and a final extension at 72 °C for 5 min. The resulting PCR products were visualized by gel electrophoresis and purified using ExoSAP-IT (USB Affymetrix Corporation, Cleveland, OH, USA) following the manufacturer’s instructions. All PCR products were sent to the Genomics Core Facility at the Pennsylvania State University for sequencing purposes. TEF regions were sequenced using the same primers used for PCR amplification. The reverse strands were sequenced only if ambiguous nucleotides were noted in the forward strands. For the IGS region additional primers were used for sequencing, other than the ones used for amplification: PN22 (5’-CAAGCATATGACTACTGGC-3’) [65], CNSa (5’-TCTCATRTACCCTCCGAGACC-3’) [66], iNL11 (5’-AGGCTTCGGCTTAGCGTCTTAG-3’) [66], U: 49-67 (5’-AATACAAGCACGCCGACAC-3’) [65] and PNFo (5-CCCGCCTGGCTGCGTCCGACTC-3’) [67]. For comparative purposes, TEF and IGS sequences of all clonal lineages of *Foc* described by Fourie et al. [10] and additional TEF sequences of *Foc* used by O’ Donnell et al. [66] were incorporated into the analyses. Sequenced products were edited and aligned using Geneious software v 7.1.4 (Biomatters, Auckland, NZ, USA). Phylogenies based on maximum likelihood (ML) and maximum parsimony (MP) methods were inferred for the different datasets using GARLI v. 2.01 [68] on CIPRES Science Gateway-website (http://www.phylo.org/portal2/login) and MEGA v. 6.0 [69]. Clade support was assessed by the bootstrap method with 1000 replications. For ML analysis, the general-time-reversible model with a proportion of invariant sites and gamma distributed rate heterogeneity (GTR + G + I) was selected as the model for nucleotide substitution. Datasets were rooted with *Fusarium* sp. NRRL 25184 and *Fusarium commune* NRRL 31076. All sequences used in the phylogenetic analyses were deposited in the GenBank database under accession numbers detailed in the Supplementary Materials, Table S2.

### 4.4. Identification of Mating-Type Idiomorphs

Mating type idiomorphs were determined using specific markers used by Arie et al. [70], Fourie et al. [10], and Steenkamp et al. [71]. The MAT1-1 idiomorph was amplified with the Falpha 1-F (5′-CGGTCAYGAGTATCTTCCTG-3′) and the Falpha 2-R (5′-GATGTAGATGGAGGGTTCAA-3′) primers. Similarly, the MAT1-2 idiomorph was amplified using the forward FF1 (5’-GTATCTTCTGTCCACCACAG-3′) and reverse Gfmat2c (5′-AGCGTCATTATTCGATCAAG-3′) primers. The cycling conditions for MAT1-1 amplification were as follows: Initial denaturation at 95 °C for 15 min; 35 cycles at 94 °C for 1 min, 55 °C for 30 s, 72 °C for 1 min and a final extension at 72 °C for 10 min. The cycling conditions for MAT1-2 were initial denaturation at 95 °C for 2 min, followed by 35 cycles of denaturation at 92 °C for 30 s, 54 °C for 40 s, 72 °C for 2 min, and a final extension at 72 °C for 7 min. The amplified products were resolved by electrophoresis using 1% agarose gel, stained with EZ-vision (Denville Scientific), and visualized under UV light. Amplicon sizes were estimated using a 100-bp ladder as reference.

### 4.5. Vegetative Compatibility Group Analyses

Nitrate non-utilizing (nit) mutants of 23 selected isolates, representative of five provinces, were at 27 °C. Colony mutants with a weak growth and no aerial mycelium were further characterized as *nit*-1, *nit*-3, or *nit*-M on media containing one of three different sources of nitrogen [72,73]. *Nit*-mutants of a given isolate were paired to identify self-incompatibility. All isolates were paired with nit-mutants from known testers VCGs 0120, 0123 and 01213, obtained from the Fusarium Research Center (FRC) at the Pennsylvania State University, University Park, PA, USA. VCG complementation was considered positive when the pairing of two nit-mutants resulted in dense aerial growth at the contact zone in minimal medium [72].

### 4.6. Pathogenicity Tests

A total of 45 isolates, selected to represent all sampling sites, were tested for their pathogenicity towards Cavendish ‘Williams’, and 14 isolates against a ‘Bluggoe’ (known in Ecuador as ‘4 Filos’) and ‘Gros Michel’ banana plants. We employed the pot-culture inoculation method described by Costa et al. [32], and an infested soil method using a cornmeal-sand mixture (CMS) for increasing *Foc* inoculum [33,74]. Tissue-cultured banana plants, obtained from a banana nursery in Florida, were acclimatized and transferred to plastic bags with autoclaved soil until they reached 30 cm in height. Plants were then inoculated with 200 g of the colonized CMS (170 g of sand, 30 g of corn meal, and 20 mL of water) with an estimated concentration of 1.0 × 10^6^ cfu/g of soil. The three cultivars aforementioned were used for race differentiation, where isolates causing wilting on ‘Gros Michel’ would be recognized as race 1, on ‘Bluggoe’ as race 2, and on Cavendish ‘Williams’ as race 4. Experiments were carried out in a greenhouse at the Pennsylvania State University, University Park, PA, USA, under semi-controlled conditions (28 °C, 80% relative humidity and 12 h light), following a completely randomized design with six replicates per treatment. The incidence of the disease (the number of plants affected per treatment) was rated 45 days after inoculation. Internal symptoms (rhizome discoloration) were assessed by visual inspection of dissected banana rhizomes, based on the rating scale proposed by Li et al. [75] where, 0 = no symptoms, 1 = 1–20% of necrosis 2 = 21–40% of necrosis, and 3 = >40% of discolored rhizome. Disease indexes were calculated according to McKinney using the formula DI = Sum of numerical ratings/Total number of plants observed) × (100/Maximum category value) [40]. In order to check for infection, at the end of the experiment, a random banana plant per treatment was selected for the isolation and visually confirmation of *F. oxysporum* based on morphological characters. At the end, all material derived from these experiments was destroyed properly. Reference *Foc* strains O-2052 (VCG01213) and O-1968 (VCG0123) were used as positive controls for TR4 and Race1/2 inoculation experiments.

## Figures and Tables

**Table 1:** Vegetative compatibility testing after complementation with selected isolates of *Fusarium oxysporum* associated to Fusarium wilt of banana from Ecuador. The strength of complementation is indicated by “+++”, strong complementation; “++”, moderate complementation; and “+”, weak complementation. “−“, no complementation.

Isolate Code	VCG 0120	VCG 0123	VCG 01213
EC4-E-GM1	++	−	−
EC10-E-GM2	++	−	−
EC12-E-GM1	+++	−	−
EC15-E-GM1	+++	−	−
EC18-E-GM1	+++	−	−
EC2-M-GM2	−	−	−
EC11-M-GM2	−	−	−
EC18-M-GM2	+++	−	−
EC29-M-GM1	++	−	−
EC32-M-GM2	+++	−	−
EC44-M-GM2	+++	−	−
EC51-M-GM3	+	−	−
EC8-G-GM1	+++	−	−
EC9-G-GM3	−	−	−
EC14-G-GM1	+++	−	−
EC21-G-GM3	−	−	−
EC23-G-GM1	+++	−	−
EC24-G-GM3	++	−	−
EC25-G-GM1	++	−	−
EC32-G-GM1	+	−	−
EC28-LR-GM1	+	−	−
EC3-O-GM2	+++	−	−
EC5-O-GM2	−	−	−

## Supplementary Materials

The following are available online at https://www.mdpi.com/2223-7747/9/9/1133/s1, Figure S1: Best ML tree inferred from TEF sequences of *Foc* population of Ecuador and other *F. oxysporum* isolates associated to banana; Figure S2: Polymerase chain reaction (PCR) analysis showing the presence of MAT1-2 idiomorph on *Foc* population of Ecuador; Table S1: List of *Fusarium oxysporum* isolates associated with banana used in this study; Table S2: GenBank accession numbers of *Foc* and other *F. oxysporum* isolates used for phylogenetic analyses.
