# Supplementary material for: Genetic Diversity of Fusarium oxysporum f. sp. cubense, the Fusarium Wilt Pathogen of Banana, in Ecuador
Source: Plants (Basel). 2020 Sep 1;9(9):1133. doi: 10.3390/plants9091133 (PMC7570379; doi:10.3390/plants9091133)
Supplement: Supplementary file 1 [file plants-09-01133-s001.zip › Figure S2.pdf]

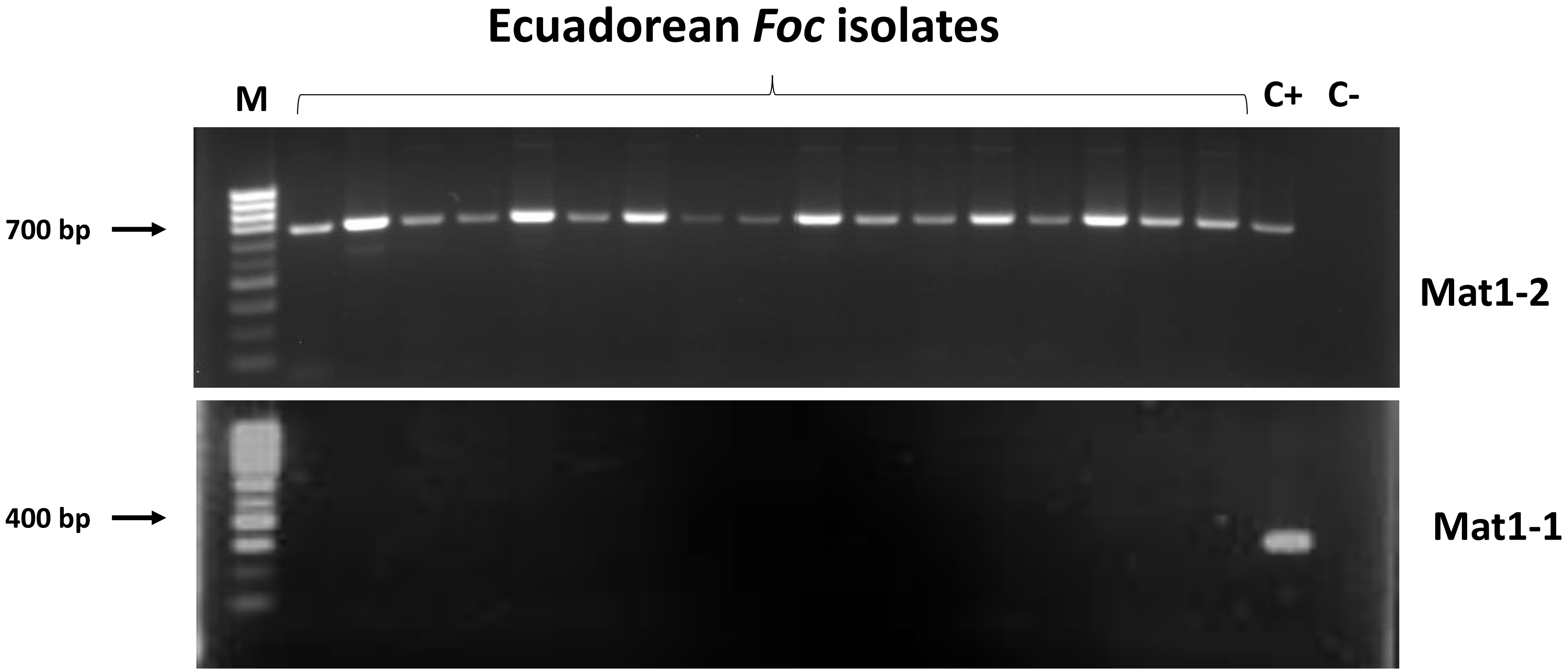

Figure S2. Polymerase chain reaction (PCR) analysis showing the presence of MAT1-2 idiomorph amplified with the specific primers FF1 and Gfmat2c on representatives *Foc* isolates of Ecuador. PCR product visualization was carried out following electrophoresis in a 0,8% agarose gel.
