# Supplementary figures and images for: Genetic Diversity of Fusarium oxysporum f. sp. cubense, the Fusarium Wilt Pathogen of Banana, in Ecuador
Source: Plants (Basel). 2020 Sep 1;9(9):1133. doi: 10.3390/plants9091133 (PMC7570379; doi:10.3390/plants9091133)

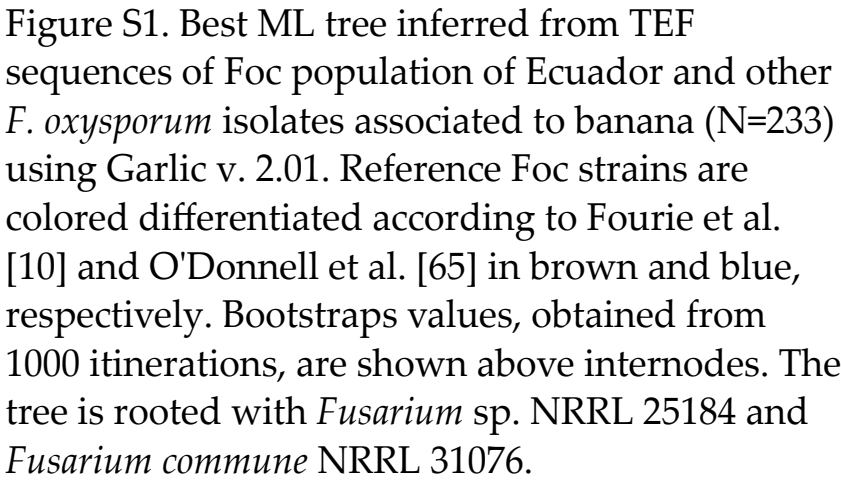

Supplement: Supplementary file 1 [file plants-09-01133-s001.zip › Figure S1.pdf]
